# Role of Intracellular Na^+^ in the Regulation of [Ca^2+^]_i_ in the Rat Suprachiasmatic Nucleus Neurons

**DOI:** 10.3390/ijms20194868

**Published:** 2019-09-30

**Authors:** Ruo-Ciao Cheng, Pi-Cheng Cheng, Yi-Chi Wang, Rong-Chi Huang

**Affiliations:** 1 Department of Physiology and Pharmacology, College of Medicine, Chang Gung University, Tao-Yuan 33302, Taiwan; 2 Healthy Aging Research Center, Chang Gung University, Tao-Yuan 33302, Taiwan; 3 Neuroscience Research Center, Chang Gung Memorial Hospital, Linkou Medical Center, Tao-Yuan 33305, Taiwan

**Keywords:** Ca^2+^, Na^+^, Na^+^/K^+^-ATPase, Na^+^/Ca^2+^ exchanger, mitochondria, suprachiasmatic nucleus

## Abstract

Transmembrane Ca^2+^ influx is essential to the proper functioning of the central clock in the suprachiasmatic nucleus (SCN). In the rat SCN neurons, the clearance of somatic Ca^2+^ following depolarization-induced Ca^2+^ transients involves Ca^2+^ extrusion via Na^+^/Ca^2+^ exchanger (NCX) and mitochondrial Ca^2+^ buffering. Here we show an important role of intracellular Na^+^ in the regulation of [Ca^2+^]_i_ in these neurons. The effect of Na^+^ loading on [Ca^2+^]_i_ was determined with the Na^+^ ionophore monensin and the cardiac glycoside ouabain to block Na^+^/K^+^-ATPase (NKA). Ratiometric Na^+^ and Ca^2+^ imaging was used to measure the change in [Na^+^]_i_ and [Ca^2+^]_i_, and cell-attached recordings to investigate the effects of monensin and ouabain on spontaneous firing. Our results show that in spite of opposite effects on spontaneous firing and basal [Ca^2+^], both monensin and ouabain induced Na^+^ loading, and increased the peak amplitude, slowed the fast decay rate, and enhanced the slow decay phase of 20 mM K^+^-evoked Ca^2+^ transients. Furthermore, both ouabain and monensin preferentially enhanced nimodipine-insensitive Ca^2+^ transients. Together, our results indicate that in the SCN neurons the NKA plays an important role in regulating [Ca^2+^]_i_, in particular, associated with nimodipine-insensitive Ca^2+^ channels.

## 1. Introduction

The central clock in the hypothalamic suprachiasmatic nucleus (SCN) controls circadian rhythms in mammals [1]. The SCN neurons express higher daytime spontaneous firing rate [2,3,4,5], [Ca^2+^]_i_ [6,7,8], Na^+^/K^+^-ATPase (NKA) and Na^+^/Ca^2+^ exchanger (NCX) activity [9,10], cytochrome oxidase activity [11], and glucose uptake [12,13]. Intracellular Ca^2+^ is essential to the proper functioning of the SCN, and its homeostasis depends on various Ca^2+^ handling systems, including those involved in mediating Ca^2+^ entry (voltage- and receptor-operated Ca^2+^ channels), extrusion (plasmalemmal Na^+^/Ca^2+^ exchanger (NCX) and Ca^2+^-ATPase), and buffering (Ca^2+^ binding proteins, endoplasmic reticulum, and mitochondria).

Previous studies have established the critical role of transmembrane Ca^2+^ flux in regulating clock genes expression, glutamate-induced phase shifts, and neuropeptide release (see [10] and references therein). To investigate the regulation of Ca^2+^ homeostasis in the SCN neurons, we recently reported that the SCN expresses NCX1 and NCX2, with NCX1 distributed in the whole SCN, and NCX2 restricted to the retinorecipient ventral SCN [10]. We showed that the clearance of Ca^2+^ following high (50 mM) K^+^-induced Ca^2+^ transients involves NCX and mitochondria, with NCX mediating fast Ca^2+^ decay and mitochondria regulating slow Ca^2+^ decay and basal [Ca^2+^]_i_. In contrast, blockade of plasmalemmal and sarco(endo)plasmic reticulum Ca^2+^-ATPase, ryanodine receptors, or IP3 receptors had little effect on the decay kinetics [10]. Most recently, we further showed that while NCX rapidly clears Ca^2+^ entry via both nimodipine-sensitive and -insensitive Ca^2+^ channels, mitochondria preferentially buffer Ca^2+^ entry via nimodipine-insensitive N-, P/Q-, and most likely also T-type Ca^2+^ channels [14].

As intracellular Na^+^ regulates both the plasmalemmal NCX as well as mitochondrial Ca^2+^ buffering via the mitochondrial NCX (NCLX) (for review, see [15,16]), it could play a potent role in regulating Ca^2+^ homeostasis in the SCN. In this study we investigated the role of intracellular Na^+^ in the regulation of [Ca^2+^]_i_ in the rat SCN neurons. Our results indicate that intracellular Na^+^ loading slows the rate of fast Ca^2+^ decay by inhibiting NCX-mediated Ca^2+^ extrusion, and enhances the slow decay phase, most likely by compromising mitochondrial Ca^2+^ buffering. Thus, via controlling intracellular Na^+^ the NKA plays an important role in regulating [Ca^2+^]_i_ in the SCN neurons.

## 2. Results

### 2.1. Monensin Effects

**Figure 1:**
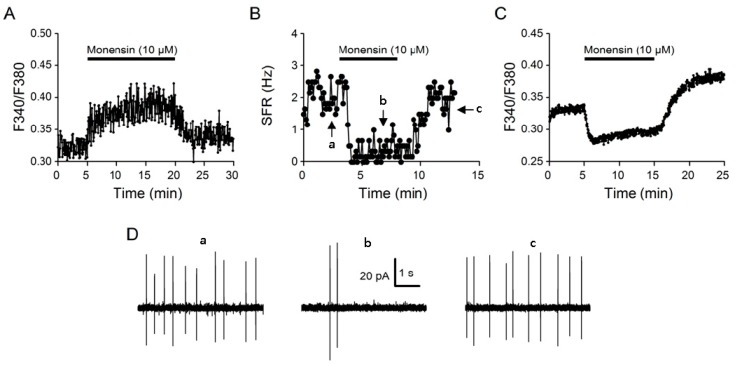
Monensin effects on [Na^+^]_i_ (**A**), spontaneous firing rate (SFR) (**B**), and [Ca^2+^]_i_ (**C**) from cells in reduced SCN preparations. Representative experiments showing the effect of 10 µM monensin on [Na^+^]_i_ (an average of 13 cells) (**A**) and [Ca^2+^]_i_ (an average of 16 cells) (**C**). (**B**) A representative cell showing the inhibitory effect of 10 µM monensin on the spontaneous firing rate. The arrows mark where the 6-s action current traces (a,b,c) plotted in (**D**) were taken from. On average, monensin inhibits the spontaneous firing rate from 3.8 ± 0.3 Hz (*n* = 15) to 0.6 ± 0.2 Hz (*n* = 15; *P* < 0.0001; paired *t*-test). (**D**) Three 6-s action current traces (a,b,c) recorded with the cell-attached technique to show the effects of 10 µM monensin on the firing pattern.

To investigate the role of intracellular Na^+^ in regulating [Ca^2+^]_i_, we first determined the effects of the Na^+^ ionophore monensin on [Ca^2+^]_i_ in the SCN neurons. In our previous studies, monensin at a concentration of 10 µM increases [Na^+^]_i_ and activates Na^+^/K^+^-ATPase (NKA) to hyperpolarize the resting membrane potential [17], and at a concentration of 1 µM also increases [Na^+^]_i_ to allow for Ca^2+^ uptake via Na^+^/Ca^2+^ exchanger (NCX) in response to the removal of external Na^+^ [10]. Figure 1 shows three representative experiments to demonstrate the effect of 10 µM monensin on [Na^+^]_i_, spontaneous firing, and [Ca^2+^]_i_. The addition of 10 µM monensin increased [Na^+^]_i_ (Figure 1A) and inhibited spontaneous firing (Figure 1B,D), confirming our previous finding that 10 µM monensin increases [Na^+^]_i_ and activates NKA to hyperpolarize the resting membrane potential [17]. Monensin also lowered basal [Ca^2+^]_i_ (Figure 1C), apparently as a result of membrane hyperpolarization and firing inhibition.

**Figure 2:**
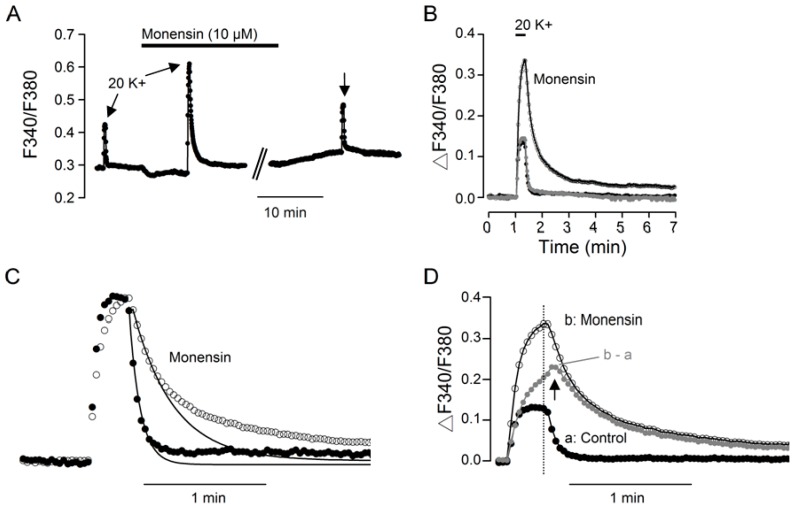
Monensin effects on the 20 K^+^-evoked Ca^2+^ transient. (**A**) A representative experiment showing the reversible effect of 10 µM monensin on the averaged Ca^2+^ response (*n* = 13 cells) to 20 mM K^+^ for 20 s. (**B**) Superimposition of Ca^2+^ transients evoked before (dark trace), during (open circle trace), and after (grey trace) the application of 10 µM monensin. (**C**) Normalized Ca^2+^ transients in the absence (filled circle trace) and presence (open circle trace) 10 µM monensin for curve fitting to obtain the fast decay time constants. The smooth curves through the data points were calculated with a fast time constant of 5.4 s and 19.8 s for control and monensin, respectively. (**D**) Superimposition of Ca^2+^ transients in control (a, dark filled circle trace) and in the presence of 10 µM monensin (b, open circle trace), as well as the digitally subtracted monensin-enhanced Ca^2+^ transient (b – a, grey filled circle trace). Note the delayed onset of Ca^2+^ clearance (marked by arrow) with continuing increase in [Ca^2+^]_i_ before decay.

Contrary to its suppressive effect on basal [Ca^2+^]_i_, 10 µM monensin markedly enhanced the Ca^2+^ response to the application of 20 mM K^+^ solution, as exemplified by the result obtained from a representative experiment (Figure 2A). Superimposition of the Ca^2+^ response to 20 mM K^+^ before (dark trace), during (open circle trace), and after (grey trace) the application of 10 µM monensin indicates that 10 µM monensin reversibly increased the amplitude of Ca^2+^ transient and slowed the rate of Ca^2+^ clearance following the termination of 20 mM K^+^ stimulation (Figure 2B). Monensin also slowed the rate of Ca^2+^ rise. This can be seen by superimposing the normalized Ca^2+^ transients to compare their kinetics in the absence (filled circles) and presence (open circles) of monensin (Figure 2C). Note the slower rate of Ca^2+^ rise, without reaching a steady state during the 20 s stimulation, for the Ca^2+^ transient in monensin (open circles; Figure 2C,D).

The monensin-induced slowing of the Ca^2+^ decay time course can be partly accounted for by the inhibition of Ca^2+^ extrusion via NCX, as suggested by the theoretic curves fitted to the fast Ca^2+^ decay phases. The single exponential curve fitted to the fast decay phase of the Ca^2+^ transient in control is taken to represent Ca^2+^ extrusion via NCX, as it is slowed threefold by the removal of external Na^+^ to block forward NCX activity [17]. For this particular experiment, monensin slowed the fast decay, with the time constant increasing from 5.4 s in control to 19.8 s in 10 µM monensin. On average, the fast decay time constant was 6.1 ± 0.4 s (*n* = 5 experiments, 120 cells) in control and 20.8 ± 1.7 s (*n* = 5 experiments, 120 cells; *P* = 0.0009; paired *t*-test) in 10 µM monensin, respectively.

In addition, monensin also enhanced the slow Ca^2+^ decay, as can be seen by superimposing the Ca^2+^ transient in the absence (filled dark circles) and presence (open circles) of monensin (Figure 2D). The digitally subtracted Ca^2+^ transient (b – a; filled grey circles), which represents the monensin-enhanced Ca^2+^ transient, has a slow rate of Ca^2+^ rise and decay. Notably, there is a delayed onset of Ca^2+^ clearance, when [Ca^2+^]_i_ appears to increase (marked by arrow), after the termination of 20 mM K^+^ stimulation (marked by dotted line). Together, the results indicate that in addition to slowing the fast Ca^2+^ decay, monensin-induced Na^+^ loading also enhances the slow decay phase, which is thought to be associated with mitochondrial Ca^2+^ buffering [10,14]. We have also investigated the effect of 1 µM monensin, which had minimal effect on the amplitude and the fast decay phase of Ca^2+^ transients, but may slightly increase the slow decay phase [18].

### 2.2. Ouabain Effects

**Figure 3:**
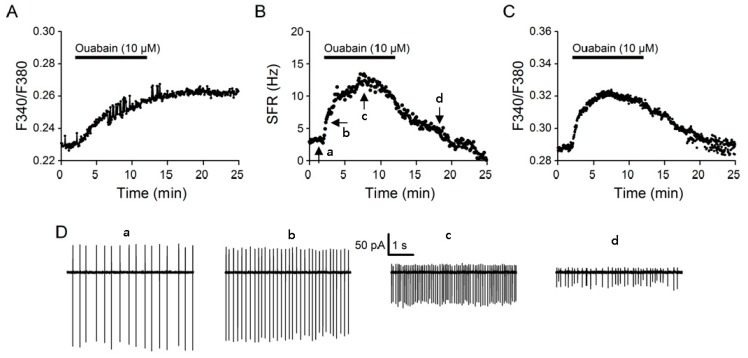
Ouabain effects on [Na^+^]_i_ (**A**), spontaneous firing rate (SFR) (**B**), and [Ca^2+^]_i_ (**C**) from cells in reduced SCN preparations. Representative experiments showing the effect of 10 µM ouabain on [Na^+^]_i_ (an average of 28 cells) (**A**) and [Ca^2+^]_i_ (an average of 21 cells) (**C**). Note the lack of recovery of [Na^+^]_i_ after the washout of ouabain. (**B**) A representative cell showing the biphasic effect of 10 µM ouabain on the spontaneous firing rate. The arrows mark where the 6-s action current traces (a,b,c,d) plotted in (**D**) were taken from. (**D**) Four 6-s action current traces (a,b,c,d) recorded with the cell-attached technique to show the effects of 10 µM ouabain on the firing pattern. Note the continuing decrease in the amplitude of spike- and afterhyperpolarization-like current. Similar results were obtained from six other cells.

We previously reported that NKA potently controls excitability and [Na^+^]_i_ in the SCN neurons [17,19]. The ability of Na^+^ loading by 10 µM monensin to inhibit forward NCX activity suggests that NKA, by regulating [Na^+^]_i_, could play an important role in the regulation of NCX activity. For comparison with the effects of monensin, ouabain at a concentration of 10 µM was also applied to determine its effects on [Na^+^]_i_, spontaneous firing, and [Ca^2+^]_i_ (Figure 3). As expected, 10 µM ouabain increases [Na^+^]_i_ (Figure 3A), spontaneous firing (Figure 3B), as well as [Ca^2+^]_i_ (Figure 3C), a result consistent with our previous findings of using K^+^-free solution to inhibit NKA [10,17,19].

However, unlike a mostly reversible effect of monensin, the effect of ouabain was virtually irreversible, as demonstrated by the lack of recovery of ouabain-induced increase in [Na^+^]_i_, which remained high after washing out ouabain for at least 15 min (Figure 3A). On the other hand, 10 min application of ouabain had biphasic effect on both spontaneous firing and [Ca^2+^]_i_, an initial increase followed by a decrease toward or even below the resting level. The trend of decrease continued after the washout of ouabain, suggesting that the ouabain effect on spontaneous firing and [Ca^2+^]_i_ is also irreversible for 15 min washout. Figure 3D shows the firing pattern in control (a, left), 1 (b, middle left) and 6 (c, middle right) min into the application, and 5 min (d, right) after the washout of ouabain. The continuing decrease in both spike- and afterhyperpolarization-like current, along with an increase and then decrease in spontaneous firing rate, suggests that depolarization block most likely caused the decrease in the firing rate [19]. Nevertheless, the biphasic change in basal [Ca^2+^]_i_ appears not to be caused by the biphasic change in firing rate, because it still occurred even in the presence of TTX to block the generation of Na^+^-dependent action potentials [18].

**Figure 4:**
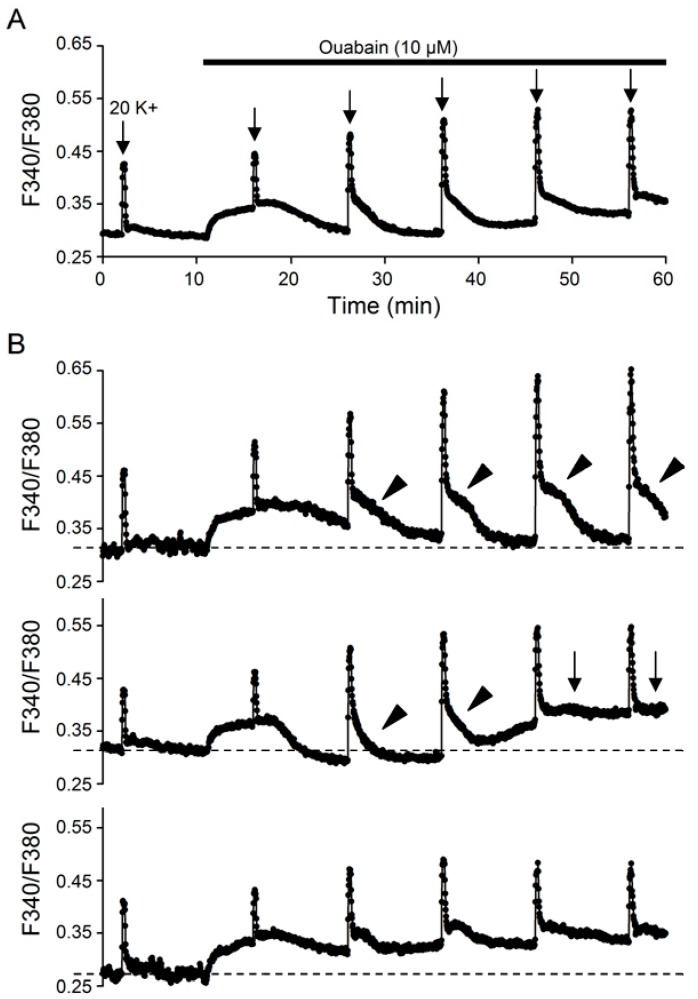
The 20 K^+^-evoked Ca^2+^ transient has plateau-like slow decay phase in ouabain. (**A**) A representative experiment to demonstrate the appearance of plateau-like slow decay phase for the 20 K^+^-evoked Ca^2+^ transient (an average of 18 cells) in the presence of ouabain. (**B**) Three representative cells to indicate the association of plateau-like slow decay phase (marked by arrowheads) with the level of basal [Ca^2+^]_i_. Note that the plateau-like slow decay phase only appears when the basal [Ca^2+^]_i_ initially raised by ouabain has began to decrease back to or even below the resting level (top, middle). For the cell in middle panel, the plateau-like slow decay phase appears and then disappears (marked by arrows) when the basal [Ca^2+^]_i_ increases again to approach the initial high level.

**Figure 5:**
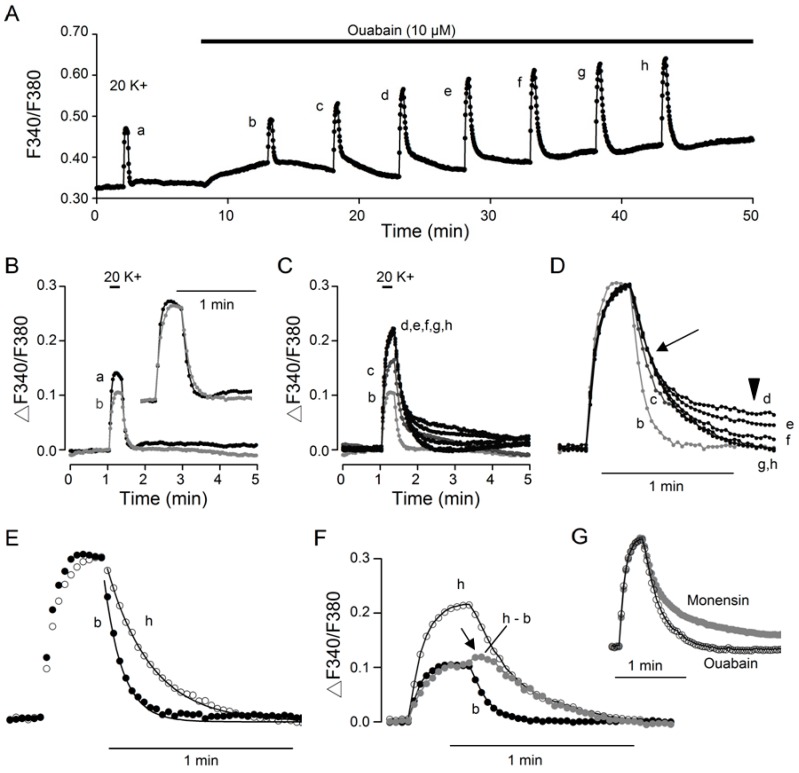
Ouabain effects on the 20 K^+^-evoked Ca^2+^ transient. (**A**) A representative experiment showing the effect of 10 µM ouabain on the averaged Ca^2+^ response (*n* = 13 cells) to 20 mM K^+^. Note the initial inhibition of the Ca^2+^ transient at 5 min into ouabain. (**B**) Superimposition of Ca^2+^ transients evoked before (a, dark trace) and at 5 min in ouabain (b, grey trace). Inset shows the normalized Ca^2+^ transients to indicate a slightly slower kinetics of Ca^2+^ transient in ouabain. (**C**) Superimposition of Ca^2+^ transients in the presence of ouabain to indicate ouabain-induced enhancement of the amplitude and slowing of the kinetics of the Ca^2+^ transient. Note the nearly identical amplitude and rising and rapid falling phases of the Ca^2+^ transients between 15 and 35 min into ouabain, indicating a steady state being reached after 15 min of ouabain application. (**D**) Normalized Ca^2+^ transients (from **C**) to indicate the ouabain-induced gradual slowing of the fast Ca^2+^ decay at 5 (b), 10 (c), and 15–35 min (d–h). Note the virtually identical fast Ca^2+^ decay (marked by arrow) but varied slow decay phase (marked by arrowhead) (d–h). The slow Ca^2+^ decay phase at 10 min (c) was truncated for better visualization. (**E**) Normalized Ca^2+^ transients at 5 min (b, filled circle trace) and 35 min (h, open circle trace) into ouabain for curve fitting. The smooth curves through the data points were calculated with a fast time constant of 6 s and 14 s at 5 (b) and 35 (h) min, respectively. (**F**) Superimposition of Ca^2+^ transients at 5 min (b, dark filled circle trace) and 35 min in ouabain (h, open circle trace), as well as the digitally subtracted ouabain-enhanced Ca^2+^ transient (h – b, grey filled circle trace). Note the delayed onset of Ca^2+^ clearance (marked by arrow). (**G**) Normalized Ca^2+^ transients in ouabain (trace h) and monensin (from Figure 2) to compare their kinetics.

As 10 µM ouabain evoked a biphasic, or even triphasic (see Figure 4B), effect on the basal [Ca^2+^]_i_, to investigate its effect on 20 K^+^-induced Ca^2+^ transients, 20 mM K^+^ solution was repetitively applied to elicit Ca^2+^ responses in the absence and then the presence of ouabain (Figure 5). The result indicates that in the presence of 10 µM ouabain, 20 K^+^-induced Ca^2+^ transient was initially reduced at 5 min (trace b), and thereafter 20 mM K^+^ solution elicited Ca^2+^ responses with progressively larger amplitude and slower decay on repetitive application to reach steady state generally after ~15 min (traces d–h) (Figure 5A). Superimposition of the Ca^2+^ transients in control (trace a) and 5 min into ouabain (trace b) indicates a smaller amplitude and a slightly slower kinetics in ouabain (Figure 5B), as can be better seen by comparing the normalized Ca^2+^ transients (inset). On the other hand, superimposition of the Ca^2+^ transients in ouabain indicates an increase in the amplitude and a slowing in the fast decay rate from 5 (trace b), 10 (trace c), to 15 (trace d) min and then leveling off thereafter to 35 (trace h) min into the application of ouabain (Figure 5C). This is best seen by superimposing the normalized Ca^2+^ transients to reveal the slowing of the fast decay phase (traces d–h) (Figure 5D). Note the virtually identical fast decay phase (marked by arrow) albeit with widely varied slow decay phase (marked by arrowhead) for traces d–h. Curve fitting to the expanded Ca^2+^ decay time course revealed a twofold increase in the time constant, from 6 s for trace b to 14 s for trace h (Figure 5E). On average, 10 µM ouabain at steady state increased the fast decay time constant from 7.2 ± 0.7 s (*n* = 7 experiments; 212 cells) to 15.0 ± 1.0 s (*n* = 7 experiments; 212 cells; *P* < 0.0001; paired *t*-test).

**Figure 6:**
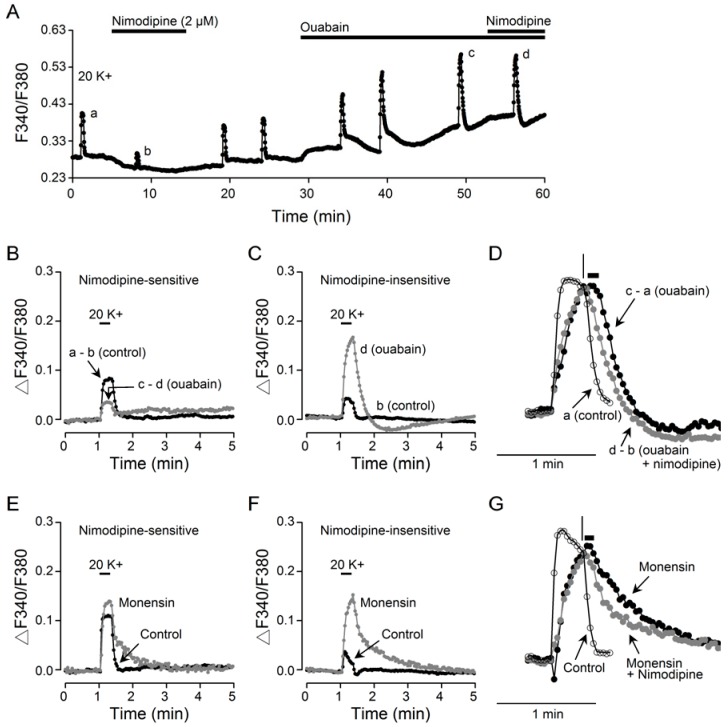
Preferential enhancement by ouabain of nimodipine-insensitive Ca^2+^ transients. (**A**) a representative experiment showing the effect of 2 µM nimodipine on 20 K^+^-induced Ca^2+^ response (an average of 13 cells) in the absence and then presence of 10 µM ouabain. (**B,C**) Superimposition of Ca^2+^ transients showing that ouabain inhibits nimodipine-sensitive (**B**) but enhances the nimodipine-insensitive (**C**) Ca^2+^ transients. (**D**) Normalized Ca^2+^ transients to show the delayed onset of Ca^2+^ clearance (marked by horizontal bar) for ouabain-enhanced Ca^2+^ transient in the absence (c – a) but not in the presence (d – b) of nimodipine. The control Ca^2+^ transient (a) was also shown to indicate the rapid Ca^2+^ decay (marked by vertical line) on termination of 20 mM K^+^ stimulation. (**E,F**) Superimposition of Ca^2+^ transients showing that monensin enhances both nimodipine-sensitive (**E**) and -insensitive (**F**) Ca^2+^ transients. (**G**) Normalized Ca^2+^ transients to show the effect of nimodipine on the delayed onset of Ca^2+^ clearance (marked by horizontal bar). The control Ca^2+^ transient was also shown to indicate the rapid Ca^2+^ decay (marked by vertical line) on termination of 20 mM K^+^ stimulation.

Similar to monensin, ouabain also enhanced the slow Ca^2+^ decay, as evidenced by superimposing the Ca^2+^ transient in the absence (filled dark circles) and presence (open circles) of ouabain, as well as the digitally subtracted Ca^2+^ transient enhanced by ouabain (h – b; filled grey circles) (Figure 5F). The ouabain-enhanced Ca^2+^ transient has a slower rate of Ca^2+^ rise and decay. In particular, there is also a delayed onset of Ca^2+^ clearance (marked by arrow) after the termination of 20 mM K^+^ stimulation. Comparison of normalized Ca^2+^ in ouabain and monensin (from Figure 2) indicates a similar rate of Ca^2+^ rise, but a slower rate of fast Ca^2+^ decay and more prominent slow decay in monensin (Figure 5G; see also Figure 6). Taken together, our results indicate that intracellular Na^+^ loading, either by monensin or ouabain, slows the fast Ca^2+^ decay but enhances the slow decay phase.

Of note, the Ca^2+^ transients evoked after ~15 min into 10 µM ouabain have virtually identical fast decay but widely varied slow decay phase, suggesting that the slow decay phase as observed in ouabain is not associated to the fast decay mediated by Ca^2+^ extrusion via NCX, the activity of which depends on both extracellular [10] and intracellular (this study) Na^+^. Furthermore, the magnitude of the slow decay appears to be associated with the levels of [Ca^2+^]_i_ when Ca^2+^ transient was elicited by 20 mM K^+^. This is evident by comparing trace d with trace f (Figure 5A), showing that trace d having lowest basal [Ca^2+^]_i_ before stimulation but largest amplitude of slow decay, whereas trace f having highest basal [Ca^2+^]_i_ with virtually no slow decay. In other words, there appears to be a negative correlation between basal [Ca^2+^]_i_ and the magnitude of the slow decay phase. This is best exemplified in a different experiment, in which Ca^2+^ transients were elicited with longer inter-application interval to allow for better visualization of the slow decay phase (Figure 4).

Figure 4A shows the average Ca^2+^ response (18 cells) to 20 mM K^+^ in the absence and presence of 10 µM ouabain to demonstrate the appearance of prominent slow decay phase in parallel to the decrease of basal level of [Ca^2+^]_i_ back to or even below the resting level, following an initial increase by ouabain. Figure 4B shows three different patterns of Ca^2+^ response in ouabain from three representative cells. For the first cell (top), after an initial increase in ouabain the basal level of [Ca^2+^]_i_ returned to near its resting level for as long as 30 min along with the appearance of prominent slow decay phase (marked by arrowheads) for the 20 K^+^-evoked Ca^2+^ transients. For the second cell (middle), after an initial increase in ouabain the basal [Ca^2+^]_i_ return to near its resting level for about 10 min only to increase back to a level similar to or higher than the initial increase by ouabain. Notably, the slow decay phase for the Ca^2+^ transient appeared (marked by arrowheads) when the basal [Ca^2+^]_i_ decreased back to close to the resting level and then disappeared (marked by arrows) altogether when the basal [Ca^2+^]_i_ increased again to the higher levels. For the third cell (bottom), there is only a small decrease in basal [Ca^2+^]_i_ following ouabain-induced initial increase and so is only a small increase in the slow decay phase.

It is beyond the scope of this study to delineate the mechanism for the parallel changes in the basal [Ca^2+^]_i_ and the appearance of plateau-like slow decay phase in ouabain. Nevertheless, it is worthy of mentioning that high K^+^-evoked Ca^2+^ transients with pronounced plateau slow decay are known to be present in peripheral neurons, with the plateau being attributed to mitochondrial Ca^2+^ uptake and release [20,21,22,23].

### 2.3. Ouabain Preferentially Enhances Nimodipine-Insensitive Ca^2+^ Rise

We previously showed that, compared to the nimodipine-sensitive Ca^2+^ response to 20 mM K^+^, the nimodipine-insensitive Ca^2+^ transient has a slower rate of Ca^2+^ rise and is preferentially buffered by mitochondria [14]. As the ouabain-enhanced Ca^2+^ transient also has a slower rate of Ca^2+^ rise, we suspected that ouabain may also preferentially potentiate the nimodipine-insensitive Ca^2+^ rise. Our result indicates that this is indeed the case (Figure 6). Figure 6A shows a representative experiment to indicate the effect of 2 µM nimodipine on 20 K^+^-induced Ca^2+^ transient first in the absence and then the presence of 10 µM ouabain. Comparison of the Ca^2+^ transients in the absence (dark traces) and presence (grey traces) of ouabain indicates that ouabain inhibited the nimodipine-sensitive Ca^2+^ transient (Figure 6B) but enhanced the nimodipine-insensitive Ca^2+^ transient (Figure 6C). On average, 10 µM ouabain inhibited the nimodipine-sensitive Ca^2+^ transient by 46% ± 2% (*n* = 6 experiments, 160 cells; *P* < 0.0001; paired *t*-test), and enhanced the nimodipine-insensitive Ca^2+^ transient by 305% ± 19% (*n* = 6 experiments, 160 cells; *P* < 0.0001; paired *t*-test). In other words, the initial inhibition of Ca^2+^ transient at 5 min into ouabain (Figure 5B) is most likely mediated by inhibition of nimodipine-sensitive component, and the gradually larger Ca^2+^ transients thereafter (Figure 5C) is mediated by the potentiation of nimodipine-insensitive component. Notably, the preferential enhancement of nimodipine-insensitive Ca^2+^ transient by inhibiting NKA with ouabain is similar to that by inhibiting mitochondrial Ca^2+^ uptake with the protonophore carbonyl cyanide-p-trifluoromethoxyphenylhydrazone (FCCP) [14], suggesting that ouabain-induced Na^+^ loading may also compromise mitochondrial Ca^2+^ uptake.

Interestingly, close inspection of the ouabain-enhanced Ca^2+^ transient in the absence (c – a; dark filled circle trace) and presence (d – b; grey filled circle trace) of nimodipine indicate that nimodipine nearly abolished the delayed onset of Ca^2+^ clearance (marked by horizontal bar, Figure 6D). The control trace (a; open circle trace) was also plotted for comparison to show the rapid decay on the termination of 20 mM K^+^ stimulation (marked by vertical line).

For comparison, we also determined the effects of 2 µM nimodipine on monensin-induced changes of 20 K^+^-evoked Ca^2+^ transients (Figure 6E–G). The result indicates that similar to the effect of ouabain (Figure 6C), monensin also markedly enhanced the nimodipine-insensitive Ca^2+^ transient (Figure 6F), by an average of 336% ± 20% (*n* = 6 experiments, 112 cells; *P* < 0.0001; paired *t*-test). Interestingly, unlike the inhibitory effect of ouabain (Figure 6B), monensin also enhanced the nimodipine-sensitive Ca^2+^ transient (Figure 6E), by an average of 42% ± 4% (*n* = 6 experiments, 112 cells; *P* < 0.0001; paired *t*-test). Furthermore, the monensin-enhanced Ca^2+^ transient also displayed delayed onset of Ca^2+^ clearance, which is also reduced by the addition of nimodipine (Figure 6G). Together the results indicate preferential enhancement of nimodipine-insensitive Ca^2+^ transients by ouabain and monensin.

**Figure 7:**
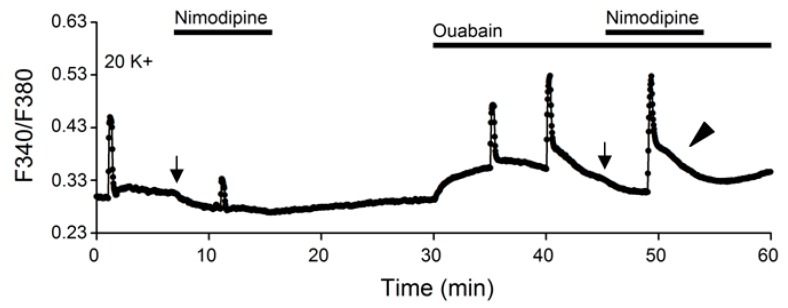
The plateau-like slow decay phase is independent of nimodipine-sensitive Ca^2+^ influx. A representative experiment to show the effect of 2 μM nimodipine on the 20 K^+^-evoked Ca^2+^ transient (an average of 12 cells). Note that the plateau-like slow decay phase (marked by arrowhead) still occurred in the presence of nimodipine to block Ca^2+^ entry via L-type Ca^2+^ channels. Arrows mark the application of 2 µM nimodipine.

Although nimodipine-sensitive Ca^2+^ influx appears to participate in the generation of the delayed onset of Ca^2+^ clearance, it is not required for the plateau-like slow decay phase in ouabain. For the experiment, nimodipine was applied when ouabain-induced increase in the basal [Ca^2+^]_i_ began to decrease back to near resting levels as exemplified in a representative experiment shown in Figure 7. As indicated, the application of nimodipine (marked by arrow) did not prevent the occurrence of plateau-like slow decay (marked by arrowhead), suggesting that the plateau-like slow decay does not depend on nimodipine-sensitive Ca^2+^ influx.

## 3. Discussion

This study demonstrates a potent regulation of [Ca^2+^]_i_ by intracellular Na^+^ in the SCN neurons. Intracellular Na^+^ loading by monensin or ouabain slows the fast Ca^2+^ decay of depolarization-evoked Ca^2+^ transients by inhibiting Ca^2+^ extrusion via the NCX, and enhances the slow decay, most likely by compromising mitochondrial Ca^2+^ buffering. Importantly, ouabain preferentially enhanced nimodipine-insensitive Ca^2+^ transients, suggesting an important role of NKA in the regulation of [Ca^2+^]_i_, in particular, associated with nimodipine-insensitive Ca^2+^ channels.

### 3.1. Monensin Effects

We show that 10 µM monensin increases [Na^+^]_i_, inhibits spontaneous firing, and lowers the basal [Ca^2+^]_i_ (Figure 1). The results confirm our previous findings that 10 µM monensin increases [Na^+^]_i_ and activates NKA to hyperpolarize the resting membrane potential [17]. Unlike its suppressive effect on the basal [Ca^2+^]_i_, monensin enhances the depolarization (20 mM K^+^)-evoked Ca^2+^ transient. The monensin-induced lowering of basal [Ca^2+^]_i_ may increase the electrochemical potential for Ca^2+^ influx to enhance Ca^2+^ response to 20 K^+^-evoked depolarization. In particular, monensin slows the fast Ca^2+^ decay, suggesting that monensin-induced Na^+^ loading inhibits forward NCX activity [10].

Furthermore, the digitally subtracted monensin-enhanced Ca^2+^ transient has a slow rate of Ca^2+^ rise and decay, with the latter characterized by a delay of continuing increase in [Ca^2+^]_i_ before the slow Ca^2+^ decay (Figure 2D). Notably, the slow kinetics and a relative insensitivity to nimodipine of the monensin-enhanced Ca^2+^ transient (Figure 6E–G) resembles that of the Ca^2+^ transient enhanced by the inhibition of mitochondrial Ca^2+^ uptake with the protonophore FCCP [14]. Together with our previous finding also suggesting an association of slow Ca^2+^ decay phase with mitochondrial Ca^2+^ uptake [10], the results suggest that monensin-induced Na^+^ loading may promote release and/or compromise mitochondrial Ca^2+^ uptake in the SCN neurons. Indeed, intracellular Na^+^ loadings have been shown to increase [Ca^2+^]_i_ by influencing mitochondrial Ca^2+^ buffering (see, for example, [24,25]).

In contrast to the marked effects of 10 μM monensin on the Ca^2+^ transient, 1 μM monensin had minimal effect on the amplitude and the fast decay phase of Ca^2+^ responses to 20 mM K^+^ solution in this study. The unexpected result is contrary to our previous finding that 1 μM monensin increases intracellular [Na^+^]_i_ to promote Ca^2+^ uptake via reverse NCX activity in response to the removal of extracellular Na^+^ [10]. One likely explanation is that the local Na^+^ concentration around the NCX might not be high enough to significantly inhibit forward NCX activity.

Nevertheless, it should be noted that monensin also alkalinizes intracellular pH, which could potentially alter [Ca^2+^]_i_ in view of our recent finding showing that intracellular pH also plays a role in the regulation of [Ca^2+^]_i_ in rat SCN neurons [26].

### 3.2. Ouabain Effects

Contrary to the suppressive effect of monensin, inhibition of NKA with 10 µM ouabain increases both spontaneous firing and basal [Ca^2+^]_i_ (Figure 3). The ouabain-induced increase in the basal [Ca^2+^]_i_ is most likely a result of membrane depolarization and increase of spontaneous firing, as has been previously shown that the blockade of NKA with K^+^-free solution reversibly depolarizes the membrane potential and increases spontaneous firing as well as [Ca^2+^]_i_ in the rat SCN neurons [10,19]. However, the mechanisms underlying the delayed decrease toward or even below the resting firing rate and [Ca^2+^]_i_, following an initial increase in response to ouabain, appear to be different. The nature of changes in the spike waveform suggests that the ouabain-induced delayed decrease in spontaneous firing is most likely mediated by depolarization block [19]. In contrast, the ouabain-induced delayed decrease in the basal [Ca^2+^]_i_ still occurred with TTX blocking the generation of Na^+^-dependent action potentials, suggesting factors other than the changing firing rate might be responsible (see below).

Nevertheless, ouabain is similar to monensin in its effects on the 20 K^+^-evoked Ca^2+^ transient, i.e., increasing the amplitude and slowing the kinetics of Ca^2+^ transients, except for its initial inhibition of the nimodipine-sensitive Ca^2+^ transient. Our results indicate that the inhibition of NKA with 10 µM ouabain increases basal [Ca^2+^]_i_ and inhibits 20 K^+^-induced Ca^2+^ transients at 5 min into ouabain, and then gradually increases the amplitude and slows the kinetics of Ca^2+^ transients, on top of delayed decrease in basal [Ca^2+^]_i_, to reach steady state after ~15 min. Notably, the time course of ouabain-induced changes in the Ca^2+^ transients is in parallel to that of ouabain-induced Na^+^ loading, suggesting that the gradual increase in cytosolic Na^+^ and decrease in the transmembrane Na^+^ gradient bring about these changes.

First, ouabain slows the rate of fast decay by approximately twofold (Figure 5E), indicating that ouabain-induced Na^+^ loading inhibits forward NCX activity. Second, similar to the monensin-enhanced Ca^2+^ transient, the slow decay of the digitally subtracted ouabain-enhanced Ca^2+^ transient is also characterized by a delay of slightly elevated [Ca^2+^]_i_ before the slow Ca^2+^ decay (Figure 5F). Third, ouabain-enhanced Ca^2+^ transient is mostly mediated by Ca^2+^ entering the nimodipine-insensitive Ca^2+^ channels (Figure 6C), reminiscent of the FCCP-enhanced Ca^2+^ transient by inhibiting mitochondrial Ca^2+^ uptake [14], suggesting that ouabain-induced Na^+^ loading also compromises mitochondrial Ca^2+^ uptake, most likely mediated by acting on mitochondrial NCLX. The monensin-enhanced Ca^2+^ transient is also mostly mediated by Ca^2+^ entering nimodipine-insensitive Ca^2+^ channels (Figure 6F), suggesting that similar mechanisms may be at work for both ouabain and monensin. Nevertheless, the reason is not known for the suppressive effect of ouabain, as opposed to the potentiating effect of monensin, on the nimodipine-sensitive Ca^2+^ transient. Further work is needed to elucidate the mechanisms for the opposite effect of monensin and ouabain on the nimodipine-sensitive Ca^2+^ rise.

Interestingly, our result also indicates a correlation between the level of basal [Ca^2+^]_i_ and the appearance of plateau-like slow decay phase in ouabain. While it remains to be determined the mechanism for the parallel changes in ouabain-induced delayed lowering of the basal [Ca^2+^]_i_ and the appearance of plateau-like slow decay phase, the observation is consistent with mitochondrial uptake and release of Ca^2+^. It is likely that the ouabain-induced delayed decrease, after an initial increase in the basal [Ca^2+^]_i_, represents mitochondrial uptake of Ca^2+^, and the plateau-like slow decay phase involves mitochondrial release of Ca^2+^ via the mitochondrial NCLX in the presence of ouabain-induced Na^+^ loading. This is reasonable, because the prominent plateau-like slow decay phase has been observed in peripheral neurons in response to large Ca^2+^ loading evoked by high K^+^ stimulation, with the plateau being attributed to mitochondrial uptake and release of Ca^2+^ [20,21,22,23].

### 3.3. Functional Implicationss

The ability of NKA, via controlling [Na^+^]_i_ and transmembrane Na^+^ gradient, to regulate NCX activity suggests an important role of NKA in the regulation of Ca^2+^ homeostasis in the SCN neurons. In particular, the diurnal rhythm in both NKA and NCX activity [9,10] suggests a concerted action of NKA and NCX, most likely, to help regulate the diurnal increase in [Ca^2+^]_i_ [6,7,8]. In what way does NKA interact with NCX to bring about their regulation of [Ca^2+^]_i_ should be an interesting issue for future study. Nevertheless, the lack of effect of 1 µM monensin on the fast Ca^2+^ decay (this study), in spite of its ability to increase [Na^+^]_i_ in the SCN neurons as shown in our previous study [10], suggests that the submembrane concentration of Na^+^ around the NCX may be tightly regulated by NKA. This is very likely, as functional linkage between NCX and NKA has been shown in cardiac muscle cells (for review, see [27]).

On the other hand, the selective potentiation of nimodipine-insensitive Ca^2+^ transients suggest that NKA also regulates mitochondrial buffering of Ca^2+^ entering the nimodipine-insensitive Ca^2+^ channels [14]. Again, in what way does the NKA interact with mitochondria to bring about such regulation should also be an interesting issue for future study. In this context, it is worthy of noting that the plateau-like slow decay phase only appears after ouabain-induced increase of basal [Ca^2+^]_i_ has began to decrease back to or even below the resting level, as if ouabain-induced increase in basal [Ca^2+^]_i_ actually promoted mitochondrial Ca^2+^ uptake to bring it down. If this is the case, then a functional linkage between NKA and mitochondria should play an important role in regulating Ca^2+^ entering the nimodipine-insensitive Ca^2+^ channels. Furthermore, as Ca^2+^ entering mitochondria is known to activate dehydrogenase to increase oxidative phosphorylation [28], our results also suggest that NKA, via regulating mitochondrial Ca^2+^ uptake, may also play a role in regulating energy metabolism.

The ability of NKA to regulate [Ca^2+^]_i_ as presented in this study suggests a mechanism how metabolic stress such as glucose shortage could alter the circadian functioning of the SCN (for review, see [29,30]). Metabolic perturbation could inhibit NKA to increase [Na^+^]_i_ in the SCN neurons [17] and the resulting alterations of [Ca^2+^]_i_ as presented in this study could potentially alter the clock function. Nevertheless, the NKA-mediated [Ca^2+^]_i_ disturbance as a result of metabolic inhibition should occur in all SCN neurons as evidenced by our previous observation that cyanide inhibition of mitochondrial respiration increase intracellular Na^+^ in all SCN neurons [17]. This is in contrast to the ATP-sensitive K^+^ channel, which appears to act as glucosensor and is preferentially expressed in the arginine-containing neurons in the dorsomedial region of the SCN [31]. Further work is warranted to determine whether glucose shortage could indeed alter [Na^+^]_i_ and [Ca^2+^]_i_ associated with metabolic inhibition of the NKA.

## 4. Materials and Methods 

### 4.1. Hypothalamic Brain Slices and Reduced SCN Preparations 

All experiments were carried out according to procedures approved by the Institutional Animal Care and Use Committee of Chang Gung University (CGU106-084, 1 December 2017). Sprague–Dawley rats (18–24 days old) were kept in a temperature-controlled room under a 12:12 light:dark cycle (light on 0700–1900 hr). Lights-on was designated Zeitgeber time (ZT) 0. For daytime (ZT 4–11) and nighttime (ZT 13–20) recordings, the animal was killed at ZT 2 and ZT 10, respectively. Hypothalamic brain slices and reduced SCN preparations were made as described previously [10,14]. An animal of either sex was carefully restrained by hand to reduce stress and killed by decapitation using a small rodent guillotine without anaesthesia, and the brain was put in an ice-cold artificial cerebrospinal fluid (ACSF) prebubbled with 95% O_2_–5% CO_2_. The ACSF contained (in mM): 125 NaCl, 3.5 KCl, 2 CaCl_2_, 1.5 MgCl_2_, 26 NaHCO_3_, 1.2 NaH_2_PO_4_, 10 glucose. A coronal slice (200–300 µm) containing the SCN and the optic chiasm was cut with a DSK microslicer DTK-1000 (Ted Pella, Redding, CA, USA), and was then incubated at room temperature (22–25 °C) in the incubation solution, which contained (in mM): 140 NaCl, 3.5 KCl, 2 CaCl_2_, 1.5 MgCl_2_, 10 glucose, 10 HEPES, pH 7.4, bubbled with 100% O_2_.

For electrical recordings and fluorescent Ca^2+^ and Na^+^ imaging, a reduced SCN preparation was obtained by excising a small piece of tissue (circa one-ninth the size of SCN) from the medial SCN using a fine needle (Cat no. 26002-10, Fine Science Tools, Foster City, CA, USA), followed by further trimming down to 4–10 smaller pieces with a short strip of razor blade. The reduced preparation was then transferred to a coverslip precoated with poly-D-lysine (Sigma-Aldrich, St Louis, MO, USA) in a recording chamber for recording. The SCN cells of the reduced preparation could be identified visually with an inverted microscope (IX70 and IX71, Olympus, Tokyo, Japan). The preparation thus obtained allows rapid application of drugs [32] and has been used for Na^+^ and Ca^2+^ fluorescent imaging [10,17] and to demonstrate diurnal rhythms in both spontaneous firing and Na/K pump activity [9].

### 4.2. Electrical Recordings

Patch clamp recording was carried out as described previously [17]. The reduced SCN preparation was perfused with bath solution containing (in mM): 140 NaCl, 3.5 KCl, 2 CaCl_2_, 1.5 MgCl_2_, 10 glucose, 10 HEPES, pH adjusted to 7.4 with NaOH. All recordings were made with Axopatch 200B amplifier (Axon Instruments, Foster City, CA, USA) at room temperature (22–25 °C). The spontaneous firing rate was recorded in the cell-attached configuration. The patch electrode was filled with the bath solution or with the patch solution containing (in mM): 20 NaCl, 1 CaCl_2_, 2 MgCl_2_, 110 K-gluconate, 11 EGTA, 10 HEPES, 3 Na-ATP, 0.3 Na-GTP, pH adjusted to 7.3 with KOH. The spike counts, in 6-s epochs, always began only after stable recordings were made. At least one or two minutes of spontaneous firing rate were counted before the application of drugs. The signal was low-pass filtered at 1–5 KHz and digitized on-line at 2–10 KHz via a 12-bit A/D digitizing board (DT2821F-DI, Data Translation, Marboro, MA, USA) with a custom-made program written in the C Language.

### 4.3. Ca^2+^ and Na^+^ Imaging

Ratiometric fluorescence imaging was carried out as described previously [10,14]. Fluorescent Ca^2+^ and Na^+^ imaging was performed, respectively, by pre-loading the SCN cells with the Ca^2+^-sensitive fluorescent indicator Fura2-acetoxymethyl ester (Fura2-AM) [33] and the Na^+^-sensitive fluorescent indicator sodium-binding benzofuran isophthalate (SBFI-AM) [34]. The reduced SCN preparation was incubated in 10 µM Fura2-AM or 15 µM SBFI-AM in 50 µl of bath solution in the dark for 60 min at 37 °C. Incubation was terminated by washing with 6 ml of bath solution and at least 60 min was allowed for de-esterification of the dye. All imaging experiments were performed at room temperature (22–25 °C). For the experiments, the reduced SCN preparation was gently pressed on the edge against the coverslip to allow adherence of the tissue to the surface. Fluorescence signals were imaged using a charge-coupled device camera attached to an inverted microscope (Olympus IX71, Japan) and recorded with Xcellence imaging software integrated with the CellIR MT20 illumination system (Olympus Biosystems, Planegg, Germany). The system used a 150-W xenon arc burner as the light source to illuminate the loaded cells. The excitation wavelengths were 340 (± 12) and 380 (± 14) nm and emitted fluorescence was collected at 510 nm. Pairs of 340/380 nm images were sampled at 0.2 Hz for Na^+^ and 0.5 Hz for Ca^2+^. Ca^2+^ and Na^+^ levels in regions of interest (ROI) over the soma were spatially averaged and presented by fluorescence ratios (F340/F380) after background subtraction. Data were analyzed and plotted with custom-made programs written in Visual Basic 6.0 and the commercial software GraphPad PRISM (GraphPad Software, San Diego, CA, USA). Data were given as means ± SEM and analyzed with paired *t*-test.

### 4.4. Drugs

Stock solutions of nimodipine (20 mM in DMSO) and monensin (10 mM in 100% ethanol) were stored at –20 °C and were diluted at least 1000 times to reach desired final concentrations. Nimodipine was purchased from Tocris Cookson (Ellisville, MO, USA), and ouabain and monensin from Sigma-Aldrich (St Louis, MO, USA). 20 mM K^+^ solution was prepared with equal molar substitution of K^+^ for Na^+^. All solutions were adjusted to pH 7.4 before use.

## Abbreviations

FCCP	Carbonyl cyanide-p-trifluoromethoxyphenylhydrazone
NCX	Na^+^/Ca^2+^ exchanger
NKA	Na^+^/K^+^-ATPase
SCN	Suprachiasmatic nucleus
